# INTERGENERATIONAL FINANCIAL TRANSFER AND FAMILY STRUCTURE IN INDIA: EVIDENCE FROM LASI, 2017–2018

**DOI:** 10.1093/geroni/igad104.1727

**Published:** 2023-12-21

**Authors:** Varsha Nagargoje, Dilip T R, K S James

**Affiliations:** International Institute for Population Sciences, Mumbai, Mumbai, Maharashtra, India; International Institute for Population Sciences, Mumbai, Mumbai, Maharashtra, India; International Institute for Population Sciences, Mumbai, Mumbai, Maharashtra, India

## Abstract

Understanding patterns of intergenerational support is critical within the context of demographic change, such as changing family structures and population aging. Existing research has analyzed the proportion of direct financial transfers between older parents and adult children and vice-versa. This study also examined the association between family structure and the direction of financial transfer in India. From the first wave of the Longitudinal Ageing Study in India, samples of 30,147 older adults aged 60+ years whose children alive were included. Bivariate analysis and logistic regression methods opted to fulfill the study objectives. Findings showed that the proportion of children-to-parent transfers (13.1%) was triple that of parents-to-children transfers (4.4%). No rural-urban disparity existed in the overall proportion of parents-to-children financial transfers. Conversely, children-to-parent financial transfers have shown marked variation among rural and urban parts of India. Older adults living alone received the most financial support from their children. While controlling sociodemographic and economic characteristics, family characteristics like having children up to eight were significantly associated with higher parents-to-children financial transfers. However, having more than eight children did not show significant effect. Having more children significantly increases the likelihood of financial transfer from children to older parents. Older adults living alone were more likely to receive financial support from children but were less likely to provide it. Filial piety is deeply rooted in India and is reflected through the bond of reciprocity among younger generations to provide financial support to their older parents, which helps older people to maintain better economic well-being.

